# Efficacy of cholecalciferol rodenticide to control wood rat, *Rattus tiomanicus* and its secondary poisoning impact towards barn owl, *Tyto javanica javanica*

**DOI:** 10.1038/s41598-023-29499-8

**Published:** 2023-02-17

**Authors:** Ariff Ateed Mohd Noh, Abu Hassan Ahmad, Hasber Salim

**Affiliations:** 1 Barn Owl and Rodent Research Group (BORG), School of Biological Sciences, Universiti Sains Malaysia, 11800 Minden, Penang Malaysia; 2 Vector Control Research Unit (VCRU), School of Biological Sciences, Universiti Sains Malaysia, 11800 Minden, Penang Malaysia

**Keywords:** Zoology, Animal behaviour, Animal physiology, Invasive species, Calcium and vitamin D

## Abstract

Studies were conducted on the potential use of cholecalciferol as an alternative to anticoagulant rodenticides to control common rat pest in oil palm plantations, i.e., wood rats, *Rattus tiomanicus,* and the secondary poisoning impact of cholecalciferol on barn owls, *Tyto javanica javanica*. The laboratory efficacy of cholecalciferol (0.075% a.i.) was compared with commonly used first-generation anticoagulant rodenticides (FGARs): chlorophacinone (0.005% a.i) and warfarin (0.05% a.i). The 6-day wild wood rat laboratory feeding trial showed cholecalciferol baits had the highest mortality rate at 71.39%. Similarly, the FGAR chlorophacinone recorded a mortality rate of 74.20%, while warfarin baits recorded the lowest mortality rate at 46.07%. The days-to-death of rat samples was in range of 6–8 days. The highest daily consumption of bait by rat samples was recorded for warfarin at 5.85 ± 1.34 g per day while the lowest was recorded in rat samples fed cholecalciferol, i.e., 3.03 ± 0.17 g per day. Chlorophacinone-treated and control rat samples recorded consumption of about 5 g per day. A secondary poisoning assessment on barn owls in captivity fed with cholecalciferol-poisoned rats showed after 7 days of alternate feeding, the barn owls appeared to remain healthy. All the barn owls fed with cholecalciferol-poisoned rats survived the 7-day alternate feeding test and throughout the study, up to 6 months after exposure. All the barn owls did not show any abnormal behavior or physical change. The barn owls were observed to be as healthy as the barn owls from the control group throughout the study.

## Introduction

Introduction of anticoagulant rodenticides (ARs) in the 1950s marked a turning point in the agriculture industry, especially in oil palm production^1^. The efficiency of ARs in dealing with rat problems became a fundamental reason for planters to switch their interest from acute poison to ARs as a default rat control measure^2,3^. However, the challenges in response to AR application emerged a few years after AR establishment. In Malaysia, resistance of a major species of rats in oil palm, wood rats (*Rattus tiomanicus*) to control ARs was one of the major concerns among planters. The first detection of wood rat-resistance against the first-generation anticoagulant rodenticide (FGAR) warfarin was reported in the 1980s in three different localities (Klang, Teluk Intan, Renggam)^3,4^. To combat this problem, more potent ARs were used and proved to be effective to control warfarin-resistant rats^5–7^. The over-dependance on toxic ARs, especially second-generation anticoagulant rodenticides (SGARs), to combat warfarin-resistant rats rose to a more complex situation when two major concerns emerged. The first concern was that the rats were observed to have developed a resistance against some of the SGARs such as bromadiolone^8–10^ and difenacoum^8,9^ and the second concern was that ARs were found to bioaccumulate in predators that feed on rats and/or other rodent pests, leading to secondary poisoning^2,11,12^.

In 1984, cholecalciferol was developed as a rodenticide by Bell Laboratories in Wisconsin and was registered as a rodenticide the same year^13,14^. The rodenticide was developed to deal with the challenges encountered in attempts to control commensal rodents, e.g., house mice^15^, with the existing ARs. New Zealand^16,17^, several European countries^14,18^ and the United States^19,20^ are countries that have established the use of cholecalciferol as a rodenticide. Initially, application of cholecalciferol was synonymous in a mixture with coumatetralyl, especially in European countries^21^. However as of 2020, the majority of the European market has cholecalciferol only baits without coumatetralyl as synonymous mixture^22^. Cholecalciferol is consumed by humans as a dietary supplement (i.e. vitamin D) and can be found naturally in fish oils, egg yolk and milk fat^23^. However, when the compound is consumed at toxic levels, it can raise the calcium level in blood (hypercalcaemia) by absorption from bone and small intestine and causes calcification of blood vessels in vital organs such as kidneys, stomach, lungs and cardiovascular system^13,24–27^. Heart failure is usually one of the major effects to the consumer, where the mineralization causes blockage to blood vessels and eventually leads to heart failure and death^17,25^. The other mechanism of cholecalciferol is the ‘stop feed effect’ where the toxin causes the consumer to lose their appetite, eventually resulting in death, which is also one of the symptoms of hypercalcemia^17,27^.

Cholecalciferol has been proven to be very effective to control Norway rats, *Rattus norvegicus* and house rats, *Rattus rattus*^13,17^. However, there are no published studies regarding the efficacy of cholecalciferol in controlling wood rats, *R. tiomanicus* in oil palm plantations, as the application of cholecalciferol is more concentrated in urban settings and poultry farms rather than in agricultural settings. In Malaysia, barn owls *Tyto javanica javanica* have been employed as natural predators, in combination with ARs to control rats in oil palm through implementation of an Integrated Pest Management (IPM) program as part of commitment towards sustainable palm oil^2,3,28^. Since the incident of warfarin resistant rats occurred, the manufacturers came up with other initiatives by applying more toxic ARs with longer hepatic biological half-life, i.e., SGARs, and this matter led to a rise in concern of potentially secondarily poisoning barn owls^29,30^. Various studies have established the existence of secondary AR poisoning of barn owls regardless FGAR or SGAR^11,31–34^. However, with regards to cholecalciferol, there have been no studies assessing the risk of secondary poisoning on barn owl. To-date, information about secondary poisoning of the toxin is available for other non-target animal species such cats and dogs^20,35^ and other bird species such as mallard duck^13,20^, domestic chicken, canary and weka^20^.

In this study, we evaluated the laboratory efficacy of cholecalciferol and commonly used FGARs to control a major rat species in oil palm plantations, i.e., wood rats, *R. tiomanicus*. We assessed whether cholecalciferol posed a secondary poisoning risk to barn owls. We did not consider FGARs in this risk assessment as there is already published data available on this in respect to barn owls.

## Methods and materials

### Laboratory trial

The aim of this study was to evaluate the efficacy of cholecalciferol (0.075% a.i) in comparison with selected FGARs to control wild wood rats, *R. tiomanicus,* in laboratory conditions using a no-choice feeding study. The study consisted of an acclimatization period, followed by a pre-test diet intake assessment, then a 6-day (multiple rodenticide dose) test period and 21 days of post-treatment observation. The lab testing procedure followed the Malaysian Standard MS1256—Household insecticide products-rat bait—chemical, physical and biological efficacy requirements (2nd revision)^36^. The study protocol was approved by an animal ethics committee (Approval number: USM/IACUC/2020/123/1064). Additionally, this study is reported based on instruction provided by ARRIVE guidelines (PLoS Bio 8(6), e1000412,2010).

### Animal trapping and laboratory feeding test

Adult *R. tiomanicus* were live-trapped in two oil palm plantations: Felda Gunung Besout 4 and Kiara Jubli Estate located in Sungkai, Perak. Traps used were drop-door cage traps (27 × 18 × 13 cm^3^) and loose oil palm fruit was used as bait. Trapping sessions were conducted for 2 weeks and about 400 individual *R. tiomanicus* were caught. The captured rats were brought back to the laboratory in Universiti Sains Malaysia (USM) for inspection. The rats were weighed, sexed and caged individually. Only rats that weighed in the range of 85–120 g were selected for the trial. All rats sampled were subjected to an acclimatization and conditioning period for at least 14 days prior to the feeding trial. Water was given ad libitum and they were fed laboratory diet (broken corn). Rats that were not eating within a normal range (3–8 g per day) were removed from the study. A total of 160 rats that were screened as healthy and feeding normally during the acclimatization period were randomly divided into 40 rats per group (20 males and 20 females) and assigned into cholecalciferol (0.075% a.i), chlorophacinone (0.005% a.i), warfarin (0.05% a.i) and control. Aforementioned total rats covered total of four replications which were carried out in this study. During the feeding trial, each rat was offered only rodenticide bait based on the treatment assigned, with water given ad libitum. Rats in the control group were given laboratory diet. Weight of the bait was recorded before being offered to the rat, and again after 24 h and replenished with fresh bait/diet (this was repeated for six consecutive days). At the end of feeding period, the rats were maintained with laboratory diet and observed until day 21. Parameters evaluated in this study were the mortality of the rats (%), days-to-death and average consumption of the baits by rat samples. Dead rats were dissected to determine the cause of death.

### Secondary poisoning to barn owls’ assessment

The study was conducted in the barn owl aviary of the School of Biological Sciences, USM, Penang. All the barn owls used in the study were captive-bred and were about 1 year old. All the barn owls were in captivity as part of the research of introducing barn owls to the campus of the University Sains Malaysia (USM), Penang. Throughout their period in captivity, prior to the secondary poisoning study, all the barn owls were fed with healthy and rodenticide-free wild wood rats. The rats were released in a feeding arena inside the aviary to be preyed upon by the owls. The captivity procedure and protocol of the study followed the guidelines suggested by^11^. A total of eight barn owls were selected for this study. After being weighed, the owls were randomly assigned to the control group (four barn owls: two males and two females) and cholecalciferol group (four barn owls: two males and two females).

Rats that were offered to the barn owls were fed with 20 g of cholecalciferol bait (0.075% a.i) each day for 2 days in no-choice feeding. Another group of healthy rats were fed with corn and offered to barn owls of the control group. All rats had free access to water ad libitum. At the end of the feeding procedure, the poisoned rats and healthy rats were offered to barn owls according to the treatment group by placing them in the feeding arena of the aviary. The remnants of the baits left by rats were collected, weighed, and recorded.

The cholecalciferol-treated owls were offered a single poisoned rat (rat fed with cholecalciferol bait for 48 h) (i.e. day 1, day 3, day 5, and day 7) and a non-poisoned rat (i.e. day 2, day 4, and day 6) on alternate days over a 7-day feeding period, depicting a 50% exposure to rodenticide in a weeks’ diet. Control barn owls received non-poisoned rats daily throughout the 7-day duration and throughout the study. Daily behavioral observations were carried out on all birds. Position and movements of the birds in the aviary were monitored. Each bird was inspected and monitored at pre-treatment, day 1, day 3, day 5, day 7 during treatment and day 11, 12, 14, 30 post treatment to observe for any signs of cholecalciferol poisoning. The weight of each bird was recorded during initial physical inspection and the same step was repeated again after the completed feeding period. The survival and the health status of all the barn owls were assessed up to 6 months.

### Data statistical analysis

One-way ANOVA was used for data analysis. The difference in mean days taken for the treatment to kill the rat samples (days-to-death) and mean amount of bait eaten between each treatment by all rats were analyzed using one-way ANOVA. Further post-hoc Tukey HSD test was used to analyze for any significant difference between each treatment group. The amount of cholecalciferol bait consumed by rats offered to each individual barn owl was analyzed by one-way ANOVA. Lastly, weight change of barn owls after the 1-week feeding trial was analyzed by non-parametric Mann Whitney U-test.

## Results

### Rat no-choice feeding test

Results of feeding test are presented in Table 1. In general, all treatments recorded mortality rates of wood rats within the range of 71–74% within 6 to 8 days of the feeding, except warfarin. FGAR chlorophacinone showed good control on the rats with mortality recorded at 74.20%, followed by cholecalciferol with a 71.39% mortality rate. Warfarin recorded only a 46.07% mortality rate of the rat samples. Chlorophacinone took 6.53 ± 1.07 days while cholecalciferol recorded 8.40 ± 0.23 days to result in death of the rat samples. Warfarin took 8.65 ± 0.67 days to result in deaths. No mortality was detected in the control group. One-way ANOVA analysis showed that there were no significant difference in days-to-death of rats between the treatments, F(3, 69) = 1.23, p = 0.30. The rats in the warfarin treatment group consumed the highest amount of bait at an average of 5.85 ± 1.34 g/day, while rats in the chlorophacinone treatment group recorded 5.43 ± 0.44 g/day bait consumption. The control group consumed 4.95 ± 0.23 g/day, and all rats in the cholecalciferol treatment group consumed the lowest amount of bait at 3.03 ± 0.17 g/day. According one-way ANOVA analysis, daily bait consumption by rats across the treatments were significantly different, F(3, 148) = 23.84, p < 0.05.

**Table 1:** Mortality rate, mean days-to-death, and bait consumption of wood rats, *Rattus tiomanicus*, in 6-day feeding trial of selected rodenticides.

Treatments	Mortality rate (%)	Means days-to-death	Mean bait consumption (g/day)
Cholecalciferol	71.39	8.40 ± 0.23^a^	3.03 ± 0.17^a^
Warfarin	46.07	8.65 ± 0.67^a^	5.85 ± 1.34^c^
Chlorophacinone	74.20	6.53 ± 1.07^a^	5.43 ± 0.44^bc^
Control	0.00	0.00 ± 0.00^b^	4.95 ± 0.23^b^

Means in column with different letters are statistically significant different (P < 0.05) by Tukey’s test.

All the rats in anticoagulant rodenticide treatments exhibited external bleeding or hemorrhage from the mouth, eyes and ears, which were common signs before death. Upon dissection, dead rats showed internal bleeding in the body cavity and general loss of blood in organs such as lungs, liver and muscle. The rats in the cholecalciferol group did not show any signs of external bleeding throughout the experiment. The bodies of the dead rats treated with cholecalciferol bait were dissected and no symptoms of external and internal hemorrhage or bleeding were observed.

### Secondary poisoning assessment

Table 2 displays the mean weight and total cholecalciferol bait consumed by rats offered to individual barn owls. Each barn owl consumed four poisoned rats with an average body weight of 93.69 ± 3.04 g. Average total bait consumed by the rats in the 2 days no-choice feeding before being offered to barn owls was 9.88 ± 0.75 g. This average consumed in the 2 days is within the normal expected dietary consumption of 3–8 g per day. The total average bait consumed corresponding to a.i. consumed by rats per body weight was 0.080 ± 0.009 mg/g. There was no statistical significant difference (F_2,9_ = 1.49, P = 0.28) in mean total bait and a.i consumed (F_3,12_ = 2.75, P = 0.89) by rats offered to each owl according to one-way ANOVA.

**Table 2:** Mean weight and total cholecalciferol bait consumed by rats offered to individual barn owls.

Barn owl		No. rats consumed	Mean weight of rat (g)	Mean total bait consumed by rats (g)	Mean total a.i. consumed by rats per body weight (mg/g)
Code (sex)	Weight (g)				
234 (♂)	450	4	97.50 ± 6.89	10.5 ± 1.19	0.081 ± 0.007
230 (♀)	434	4	102.75 ± 7.41	7.75 ± 0.63	0.057 ± 0.004
236 (♂)	470	4	85.25 ± 3.04	11.25 ± 1.44	0.098 ± 0.011
220 (♀)	473	4	89.25 ± 3.33	10.00 ± 1.58	0.085 ± 0.016
Mean	457.00	4	93.69 ± 3.04	9.88 ± 0.75	0.080 ± 0.009

Results of the toxicity effect of cholecalciferol on barn owls fed with four poisoned rats each in the 7-day feeding period are shown in Table 3. In general, all treated owls did not show any behavioral or physical abnormality. The behavior of all the barn owls after consuming the poisoned rats were examined through their movement, especially the flying activities in the aviary. The flying activities of the barn owls after consumption of four poisoned rats did not change from the pre-treatment condition and the treated owls were as active as the barn owls from control group throughout the feeding and observation period. None of the barn owls showed any change in their feeding activities, the treated owls fed normally on the rats offered, even in the post feeding period. During the observation period, all owls spent more time on the perching point than on the ground, indicating normal barn owl behaviour. The owls did not display behaviours indicative of cholecalciferol toxicity in birds, such as described by^37^; restrained behaviour, loss of weight, and inability to fly.

**Table 3:** Toxicity effects of cholecalciferol on barn owls fed with four poisoned rats in the 7 days feeding period.

Treatments	Owl code	Sex	Initial weight	Weight at day 7 (g)	Weight change after 1 week (g)	Fate after 6 months	Toxicity symptoms
Cholecalciferol	230	F	434	439	+ 5	S	
	220	F	473	464	− 9	S	Not detected
	234	M	450	476	+ 26	S	
	236	M	470	490	+ 20	S	
Mean			456.75 ± 9.14	467.25 ± 10.81	10.50 ± 7.86	–	
Untreated	101	F	455	467	+ 12	S	
	102	F	490	500	+ 10	S	
	103	M	482	495	+ 13	S	Not detected
	104	M	481	497	+ 16	S	
Mean			477.00 ± 7.60	489.75 ± 7.65	12.75 ± 1.25	–	

*S* survive.

*All means are statistically no significant difference according to non-parametric Mann Whitney U test (P > 0.05).

The physical characteristics of the treated birds after examination were similar to the control group which were fed with healthy rats. All the barn owls gained weight during the 7-day feeding period except for barn owl code 220 which experienced a slight weight reduction compared to its initial weight. Mean weight increase of barn owls after the 1-week feeding trial was recorded at 10.50 ± 7.86 g for the cholecalciferol group while the control group owls recorded a higher weight gain at an average of 12.75 ± 1.25 g. The Mann Whitney test showed that there was no statistical difference (U (N_cholecalciferol_ = 4, N_control_ = 4) = 8.00, z = 0.00, p > 0.05) in weight change between treated and untreated owls.

All treated barn owls survived the 7-day feeding period. There were no toxicity symptoms observed during the feeding period and post-feeding period up to 6 months after the treatment as all the barn owls were observed to be as healthy as barn owls in the control group. All the barn owls were later successfully released in the university campus as part of our soft release method to introduce acclimatized barn owls.

## Discussion

The feeding test results of cholecalciferol to control wood rats, *R. tiomanicus,* showed that cholecalciferol is efficacious to control a major rat species in oil palm plantations and is comparable to other FGARs such as warfarin and chlorophacinone. Cholecalciferol is known to be effective to control Norway rats (*Rattus norvegicus*) and house mice (*Mus musculus*). Marshall^13^ reported that all tested Norway rats (n = 145) and house mice (n = 100) died after consuming cholecalciferol baits with a 750 ppm (0.075%) concentration, with an average 3.9 to 6.1 days (mice) and 3.3 to 4.7 days (Norway rats) to result in death, though the number of days of the feeding trial was not mentioned in the report. Similarly, Eason et al.^17^ reported that cholecalciferol baits at a higher concentration i.e.,0.8% (originally used to control possums, *Trichosurus vulpecula*) was effective to control Norway rats and house rats (*Rattus rattus*). The authors ran a feeding trial of choice and no-choice feeding for 2 days on 35 rats consisting of ship rats (*n* = 20) and Norway rats (*n* = 15). Mortality was recorded in 34 of the 35 rats (97%) in an average of 4 days.

Based on our observation, cholecalciferol produced similar results as chlorophacinone to control wood rats, i.e., mortality rate and days-to-death, despite cholecalciferol having a lower daily dosage consumption than chlorophacinone. However, it should be noted that the results of this study were from a no-choice feeding test, and the LD_50_ reported in this study may differ in a choice-feeding and multiple-choice feeding test. Based on the oral LD_50_ of both compounds on Norway rats, LD_50_ of cholecalciferol (43.6 mg/kg) was higher than chlorophacinone (20.5 mg/kg)^13,17,38^. This shows that the rats are more susceptible to chlorophacinone than cholecalciferol due to the lower LD_50_ value. Hence, the high concentration of cholecalciferol in formulated baits compared to other anticoagulants was necessary since only a high concentration of the compound can make the formulated rodenticide effective to control target pests^39^. Based on a publication by^40^, 1 day feeding of chlorophacinone with concentration of 0.025% was able to result in 60% mortality of mice (*n* = 20) and the mortality achieved was more than 70% after 2 days feeding of the bait. 95% mortality was achieved after 21 days of multiple feeding by the mice. According to publication results by^13^ mentioned earlier, all tested mice (n = 100) died after fed baits containing 0.075% cholecalciferol. However, a contradiction to the results reported by^13^ was reported by^41^ where 0.075% of cholecalciferol was only sufficient to kill rats but not mice. Hix^41^ stated that based on feeding trials, both rat and mice can be controlled with higher dosage at 0.4% cholecalciferol.

The poor performance by warfarin compared to cholecalciferol was expected as reflected in the mortality rate, as the mortality of rats in the warfarin treatment did not even reach half of the total sample of rats tested. Even though the amount of bait consumed per day by rats in the feeding trial was the highest among all treatments, it only resulted in 44% of mortality of the rat samples, though warfarin took a shorter time than cholecalciferol to result in mortality of rats. The poor performance of warfarin has been reported in many publications due to resistance which was recorded since the 1980s in Malaysian wood rats^3,4,42^. However, there have been no proper laboratory trials conducted in wood rats except by^43^. The researchers reported that warfarin compound was effective to control 80–95% of wood rat samples (*n* = 20) but it took 6–8 days feeding of the baits. This showed that warfarin was still effective to control wood rats at that time, as the initial reports of wood rat resistance against warfarin was only documented in 1983 and began to be considered a serious problem after 1985 when three different localities in Malaysia had the same resistance problem^4^. The results of^43^ are in contrast with our current study where the rats consumed 31.10 ± 1.96 mg/kg a day for 6 days feeding but were unable to achieve 50% mortality; indicating that the species can tolerate the toxin compared to the situation of 36 years ago. There is no warfarin species-specific LD_50_ for wood rats, but it is available for house mice and Norway rats. A higher acute oral LD_50_ of warfarin is reported for house mice at 374 mg/kg, while for Norway rats it is between 58 and 323 mg/kg, depending on the strain^29,44^. In comparison, cholecalciferol has a lower LD_50_ in mice and Norway rats at 42.5 and 43.6 mg/kg respectively, reflecting that rodents are more susceptible to cholecalciferol compared to warfarin.

In Malaysia, secondary poisoning is one of the main issues with regards to AR usage since in oil palm plantations (one of the main agriculture sectors in Malaysia), barn owls are utilized as a biological control agent in order to reduce dependency on chemical practices such as rodenticides^3^. Uncontrolled application of ARs can result in deleterious effects on barn owls as the diet of the owls are highly dependent on rats which make up about 99% of prey^2,11,45,46^. There are various reports on secondary poisoning risks of ARs on non-target animals, including barn owls (*Tyto alba*). However, there is lack of information on the effects of cholecalciferol on barn owls, despite the fact that past studies suggest that the compound is less hazardous to bird species than to some mammal species^20,29,47^. One example of a less susceptible bird species to cholecalciferol is the mallard duck, whose oral LD_50_ of cholecalciferol was as high as 2000 mg/kg^13^. The conclusion made by^13^ was later confirmed by^20^, who found that none of the six ducks tested suffered lethal effects even at a dose of 2000 mg/kg. On the other hand, Eason et al.^20^ stated that 3 out of 4 and 1 out of 4 of total domestic chickens and canaries tested died when given the same dose. Meanwhile in the same study, 10 of 16 weka (*Gallirallus australis*) which voluntarily ingested 0.1% cholecalciferol bait exhibited no signs of toxicity.

In addition to lower primary toxicity of cholecalciferol as reported in the aforementioned acute toxicity bird studies, there are published secondary toxicity studies of cholecalciferol towards a range of animals. Eason et al.^20,35^ reported that feral cats showed no signs of poisoning after being fed with poisoned-possum carcasses for 5 to 6 days. However, dogs are quite susceptible to cholecalciferol regardless of primary or secondary exposure. According to^48^, 540 g of cholecalciferol bait at 10 mg/kg and 20 mg/kg can be fatal to dogs based on feeding test conducted by the researchers on two groups of dogs (n = 2). Upon consumption of a lethal dose, dogs reportedly exhibited signs of hypercalcemia and hyperphosphatemia such as lethargy, weakness and anorexia, and had internal symptoms such as gastrointestinal hemorrhage, myocardial necrosis and mineralization of vascular walls. Studies on the effect of cholecalciferol poisoning in dogs were more recently done by^20^ where test animals were fed five poisoned-possums which were dosed with 0.8% cholecalciferol-treated cereal baits. Eason et al.^20^ additionally reported that all dogs survived and recovered gradually to pre-treatment condition without veterinary intervention.

A similar study using a lower concentration of cholecalciferol (commonly used as rodenticide) conducted by^13^ reported no sign of toxicosis observed in six beagle dogs after being fed with poisoned-carcasses of Norway rats, which died after feeding on 0.075% cholecalciferol bait prior to the feeding trial. In our present study, we let the rats voluntarily consume the 0.075% cholecalciferol bait for 2 days and recorded an average 0.08 mg/g a.i per body weight. This is far lower than the dose given by^20^ to the possums before feeding them to cat and dogs but similar to concentration used by^13^ because in this study we used cholecalciferol bait for rodents (0.075% a.i.) where the a.i concentration was ten times lower than possum baits (0.8% a.i.). The barn owls which consumed the poisoned rats did not display any signs of toxicity from secondarily consuming cholecalciferol such as typical behaviour aberration. For instance, the behaviours that are commonly observed in poisoned barn owls are less flying activity, passive manner and spending more time on the ground rather than on perching point, along with reduction of weight as stated by^11^ as symptoms of barn owls affected due to secondary poisoning of AR rodenticides.

As mentioned above, there have been assessments of secondary poisoning risks of several ARs on barn owls. Mendenhall and Pank^33^ stated that consumption of rats poisoned with SGAR compounds such as bromadiolone, brodifacoum and difenacoum by barn owls caused hemorrhaging effect after a 1-week feeding trial. Gray et al.^49^ recorded that at least one out of four barn owls did not survive the feeding test of mice poisoned with brodifacoum and difenacoum while two of four owls did not survive after consuming flocoumafen-poisoned mice. In Malaysia, Lee^32^ stated that not only SGARs such as bromadiolone, brodifacoum and flocoumafen, but FGAR compounds such as warfarin also caused high degree of toxic effects on barn owls. The author fed four barn owls with poison rats treated with SGAR bait which resulting in death of three of four owl samples after 2 weeks of the exposure, while in the same study on another group of barn owls (n = 4) fed with poison rats treated with FGAR compound, warfarin has caused death on two of four tested barn owls after 3 weeks of exposure to the poisoned rats. A study conducted by^11^ which consisted of two group of four barn owls where each group of barn owls were fed with chlorophacinone and bromadiolone treated rats. The result showed that one of four tested owl samples from each group were observed with following sign of poisoning, coarse breathing, reduce of weight and flying activity, hemorrhage at the beak and hematoma (bromadiolone) after consuming three poisoned rats in a 1 week feeding period.

The secondary toxicity effect of ARs is not only reported in barn owls, but also in other non-target raptors. Lutz^50^ recorded an increase in blood coagulation after Eurasian buzzards (*Buteo buteo*) were fed with bromadiolone-poisoned mice for 10 days. Grolleau et al.^51^ observed that 27 Eurasian buzzards (*Buteo buteo*) exhibited bleeding after feeding on bromadiolone-poisoned voles for 3 days. In the same study with mammals, 10 tested ermines (*Mustela erminea*) were observed to be bleeding after being fed with bromadiolone-poisoned vole for 3 to 5 days. Another literature revealed that an increase in blood coagulation time was reported in Eurasian buzzards after fed poisoned mice^52^. Twenty American kestrels (*Falco sparverius*) exhibited external bleeding after feeding on chlorophacinone-poisoned voles for 1 to 3 days^53^. In an extensive study by^54^ in a laboratory setting, American kestrels from two different treatments, mechanically (4 out of 15) and biologically-amended (2 out of 15) rat tissue with chlorophacinone at 0.15, 0.75 and 1.5 µg chlorophacinone/g wet weight showed overt signs of toxicity effect by chlorophacinone at necropsy such as poor grooming, pale liver, frank blood on its feather, bruise on featherless tract, pale viscera and swollen liver.

Another study by^55^ on eastern screech-owls (*Megascops asio*), which was also conducted in the same setting, the owls which fed on 25 g meatball with dosage of 7.15 to 8.49 mg/kg body weight diphacinone shown similar overt signs of toxicity effect of chlorophacinone such as frank blood, bruising, pale viscera and pooled blood in muscle. Several predators such as black kites, red kites, short toed snake-eagles, and golden eagles showed flocoumafen contamination, as reported by^56^ in an opportunistic study on carcasses. Warfarin is generally considered less hazardous to non-target animals. Minks, least weasels and dogs have recorded deaths and survivors exhibited external bleeding signs after eating prey poisoned with warfarin^57–59^.

One of the reasons our secondary poisoning assessment on barn owls did not include other ARs used in the feeding trial was due to the fact that data on the poisoning of barn owls are already established from past publications. Moreover, barn owls are a protected species in Malaysia under Act 716, Wildlife Conservation Act 2010. Thus, only a limited number of samples were permitted for this study and there was no necessity to run a higher number of samples simply to confirm known poisoning effects from other ARs. Past publications such as those mentioned above, have reported the toxic effects of ARs, both FGARs and SGARs, on non-target barn owls via secondary poisoning.

## Conclusion

Based on the results of this study, cholecalciferol is potentially a good alternative rodenticide to control rats in oil palm plantations. Cholecalciferol can be considered as a better choice compared to FGARs as from this study there were no barn owl deaths seen after consuming 4 poisoned rats over a 7-day period. A secondary poisoning assessment on barn owls fed with poisoned rats showed that after 7 days of alternate feeding, the barn owls appeared to remain healthy (based on weight and behavioural assessment) and there were no mortalities recorded throughout the study period. In current times where environmental concern is a pressing worldwide issue, practices that are less detrimental to the environment need to be a primary option for plantation operators, while also complying with standards set by the Roundtable on Sustainable Palm Oil (RSPO) and Malaysian Sustainable Palm Oil (MSPO), which encourage the concept of integrated pest management practices in plantations instead of heavy reliance on ARs that are a poisoning risk to non-target animal. The use of the less toxic cholecalciferol coupled with barn owls as biological control agents could provide a sustainable rodent management solution in plantations and the environment.

## Data Availability

The datasets used and/or analysed during the current study available from the corresponding author on reasonable request.
